# Risk factors for radiographic progression in psoriatic arthritis: subanalysis of the randomized controlled trial ADEPT

**DOI:** 10.1186/ar3049

**Published:** 2010-06-10

**Authors:** Dafna D Gladman, Philip J Mease, Ernest HS Choy, Christopher T Ritchlin, Renee J Perdok, Eric H Sasso

**Affiliations:** 1 University of Toronto, 399 Bathurst Street, Room 1E-410B, Toronto, Ontario M5T 2S8, Canada; 2 Swedish Medical Center, 1101 Madison Street, Suite 1000, Seattle, WA 98104, USA; 3 King's College, Strand, Denmark Hill Campus, London WC2R 2LS, UK; 4 University of Rochester, 601 Elmwood Avenue, Rochester, NY 14642, USA; 5 Abbott Laboratories, 100 Abbott Park Road, Abbott Park, IL 60064-3500, USA

## Abstract

**Introduction:**

To identify independent predictors of radiographic progression in psoriatic arthritis (PsA) for patients treated with adalimumab or placebo in the Adalimumab Effectiveness in PsA Trial (ADEPT).

**Methods:**

Univariate analyses and multivariate linear regression analyses assessed risk for radiographic progression (change in modified total Sharp score, ΔmTSS > 0.5) from baseline to week 24 for C-reactive protein (CRP) and other baseline variables, and for 24-week time-averaged CRP (univariate analysis only). Subanalyses determined mean ΔmTSS for CRP subgroups. Analyses were *post hoc*, with observed data.

**Results:**

One hundred and forty-four adalimumab-treated patients and 152 placebo-treated patients were assessed. Mean CRP was 64% lower by week 2 with adalimumab and essentially unchanged with placebo. Univariate analyses indicated that elevated CRP at baseline and time-averaged CRP were strongly associated with radiographic progression for placebo-treated patients but not for adalimumab-treated patients. Multivariate analysis confirmed that elevated baseline CRP was the only strong independent risk factor for radiographic progression (for CRP ≥1.0 mg/dl: odds ratio = 3.28, 95% confidence interval = 1.66 to 6.51, *P *< 0.001). Adalimumab treatment reduced risk of progression approximately fivefold. The difference between mean ΔmTSS for adalimumab versus placebo was greatest for patients with baseline CRP ≥2.0 mg/dl (-0.5 vs. 2.6).

**Conclusions:**

Systemic inflammation in PsA, as indicated by elevated baseline CRP, was the only strong independent predictor of radiographic progression. This association was observed predominantly for placebo-treated patients. Adalimumab treatment substantially reduced the overall risk of radiographic progression, and provided greatest radiographic benefit for patients with the greatest CRP concentrations at baseline.

**Trial Registration:**

Trial registration: NCT00195689.

## Introduction

Psoriatic arthritis (PsA) is an inflammatory arthritis found in up to approximately 30% of patients with psoriasis and in 0.3 to 1% of the general population [1]. PsA was previously considered a mild form of arthritis, typically less severe than rheumatoid arthritis (RA). Evidence has accumulated, however, to show that PsA is associated with substantial morbidity [2-5]. Progression of clinical and radiographic damage in PsA has been related to disease activity and severity, both at presentation and at follow-up [6]. Progressive erosive disease had been reported in more than one-half of patients with PsA and is often associated with functional impairment [2,3,7,8]. Patients with PsA are at increased risk of death compared with the general population [9], and severity of PsA at presentation is a predictor of mortality [10].

Before the advent of biologic agents, therapies were employed in PsA based on experience in RA, despite differences in the types of joint damage typical of each disease and despite the lack of evidence to support prevention of clinical or radiographic damage in PsA. Randomized controlled trials of traditional disease-modifying antirheumatic drugs for patients with PsA have not included radiographic assessments, and data from an observational study provided no evidence that disease-modifying antirheumatic drugs prevented radiographic damage in PsA [11]. In contrast, randomized controlled trials with anti-TNF agents in patients with PsA have demonstrated not only clinical efficacy, but also significant inhibition of radiographic progression [12-17].

The Adalimumab Effectiveness in PsA Trial (ADEPT) is one of the largest randomized, double-blind, placebo-controlled trials of a TNF antagonist for treatment of PsA to date. ADEPT demonstrated that 24 weeks of treatment with adalimumab improved arthritis, skin disease and quality of life, and prevented radiographic joint destruction in patients with PsA [13]. Subanalyses of ADEPT have suggested that radiographic progression was associated with several baseline factors, including elevated values of C-reactive protein (CRP), swollen joint count (SJC), and tender joint count (TJC) [14]. It is not known which factors, if any, were independently associated with radiographic progression in ADEPT.

CRP is a sensitive marker for systemic inflammation. Elevated concentrations of CRP have been associated with radiographic progression in RA [18,19]. PsA and RA have distinct types of joint pathology, however, so they do not necessarily have identical risk factors for joint damage. In addition, CRP is not always elevated in patients with clinically active arthritis. The aim of this *post hoc *analysis was therefore to extend previous studies in PsA by determining whether CRP or other factors were independent predictors of radiographic progression in ADEPT. We found that baseline CRP, as measured with a high-sensitivity assay, was the dominant independent predictor of radiographic progression, and that the relationship between CRP and radiographic progression was different for patients treated with placebo compared with adalimumab.

## Materials and methods

### ADEPT study design

ADEPT was a phase III, randomized, double-blind, placebo-controlled trial in which patients were randomized to receive subcutaneous placebo or adalimumab 40 mg every other week. Randomization was centrally stratified by methotrexate use (yes/no) and extent of psoriasis (< 3% or ≥3% body surface area (BSA)) at baseline. Radiographs of the hands and feet were obtained at baseline and week 24, and were read by two blinded readers using a modified version of the total Sharp score (mTSS) that included assessments of distal interphalangeal joints [13]. Clinical and laboratory assessments were performed throughout the study, as previously described [13].

ADEPT was conducted in accordance with the principles of the Declaration of Helsinki (2000), and the protocol was approved by the institutional review boards of the participating centers. All patients provided written informed consent before any study-related procedures were initiated [13].

### Patients

The present analysis included all patients in ADEPT for whom radiographs had been obtained at baseline and week 24. ADEPT eligibility criteria required a minimum of three tender joints and three swollen joints, active psoriatic skin lesions or a history of psoriasis, and a history of an inadequate response or intolerance to nonsteroidal anti-inflammatory drugs. Eligibility criteria are described in greater detail elsewhere [13].

### Clinical and laboratory assessments

Numbers of joints evaluated were 78 for TJC and 76 for SJC. Serum CRP was assessed at a central laboratory with a high-sensitivity assay (upper limit of normal = 0.287 mg/dl), for samples obtained at baseline and weeks 2, 4, 8, 12, 16, 20 and 24. Anti-cyclic citrullinated peptide antibodies were not measured in ADEPT.

### Predictor variables

Variables tested as risk factors for radiographic progression included the following baseline measures: treatment received (adalimumab vs. placebo), patient age, PsA disease duration, psoriasis disease duration, continuous CRP, categorical CRP (≤0.287 vs. > 0.287 mg/dl; < 1.0 vs. ≥1.0 mg/dl; < 2.0 vs. ≥2.0 mg/dl), mTSS, joint erosion (JE) score, joint space narrowing (JSN) score, SJC, TJC, body weight, methotrexate use (yes/no), and rheumatoid factor (positive/negative). For the univariate analyses only, 24-week time-averaged CRP was also tested as a predictor variable.

### Outcome measure

The outcome measure for univariate and multivariate analyses was radiographic progression, defined as a change in modified total Sharp score (ΔmTSS) > 0.5 from baseline to week 24 (yes/no). The mTSS value of 0.5 was chosen because it is an accepted threshold that has been previously used to assess radiographic progression in ADEPT [14] and early RA [20].

### Subgroup analyses

Mean ΔmTSS from baseline to week 24 was determined for patients grouped *post hoc *by baseline CRP concentration (< 1.0 vs. ≥1.0 mg/dl); by baseline CRP concentration using the following four categories: ≤0.287 mg/dl, > 0.287 to < 1 mg/dl, ≥1.0 to < 2.0 mg/dl, and ≥2 mg/dl; and by time-averaged CRP concentration each using the following four categories: ≤0.287 mg/dl, > 0.287 to < 1 mg/dl, ≥1.0 to < 2.0 mg/dl, and ≥2 mg/dl. These four categories were chosen to represent CRP concentrations that are normal (for the high-sensitivity assay employed in ADEPT), slightly elevated (or approximately normal, for nonhigh-sensitivity assays), moderately elevated, and very elevated, respectively. Mean CRP concentrations were determined for patients grouped by whether they had radiographic progression (ΔmTSS > 0.5) or not (ΔmTSS ≤0.5) from baseline to week 24.

### Cumulative probability plots

Cumulative probability plots were generated to depict ΔmTSS results for all patients by treatment arm, with separate curves for patients with baseline CRP < 1.0 mg/dl and baseline CRP ≥1.0 mg/dl.

### Statistical analyses

Summary statistics were provided for baseline variables by treatment group. The mean and standard deviation were summarized for continuous variables, and the number and percentage of patients for categorical variables.

Differences between treatment groups for the change from baseline in CRP by visit were analyzed by an analysis of variance model with treatment group and baseline methotrexate use/extent of psoriasis as factors (yes/≥3% BSA, yes/< 3% BSA, no/≥3% BSA, no/< 3% BSA). Within each CRP subgroup (≤0.287 mg/dl, > 0.287 to < 1.0 mg/dl, < 1.0 mg/dl, ≥1.0 mg/dl, ≥1 to < 2.0 mg/dl, and ≥2.0 mg/dl), differences between treatment groups were compared using an analysis of covariance model, with treatment as a factor and ranked baseline mTSS as the covariate for changes in mTSS at week 24. For each patient, time-averaged CRP (in mg/dl) was determined as the area under the curve of CRP values from baseline to week 24 (in units of mg/dl-weeks), divided by 24 weeks.

Univariate analyses were performed using logistic regression models for each treatment separately and combined to determine potential associations between radiographic progression and each predictor variable. Multivariate analysis using a logistic regression model was performed for selected baseline variables, including all that were statistically significant (*P *< 0.05) in the univariate analysis, except for JSN and JE scores (to prevent duplication with mTSS). The multivariate analysis was performed four times: once with CRP as a continuous variable, and three times with CRP as a dichotomous variable (once each at a threshold of 0.287, 1.0 and 2.0 mg/dl). The results for non-CRP risk factors are reported from the analysis with CRP as a dichotomous variable at a threshold of 0.287 mg/dl.

All statistical tests were two-sided and considered significant at α = 0.05. *P *< 0.05 was considered statistically significant. All data are observed, without imputation; except for the mean CRP values determined according to radiographic progression (yes/no), which used the last observation carried forward for missing data.

## Results

**Figure 1:**
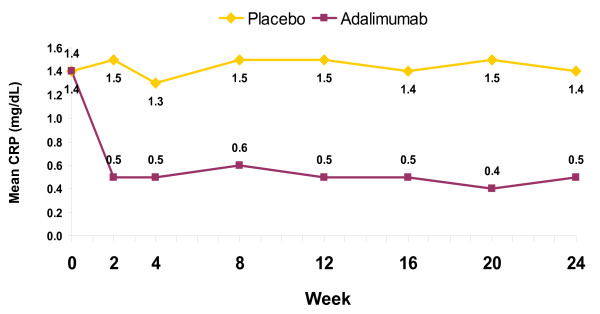
**Mean C-reactive protein concentrations over time**. Serum concentrations of C-reactive protein (CRP) were determined from baseline to week 24 for patients treated with placebo or adalimumab in ADEPT. Data are presented as mean values at each time point. Data are observed; n values at baseline/week 24 were 152/146 for placebo and 143/138 for adalimumab. *P *< 0.001 for the comparison of mean changes from baseline for adalimumab versus placebo at all time points after week 0.

This *post hoc *analysis included the 152 placebo-treated patients and 144 adalimumab-treated patients from ADEPT who had evaluable radiographs at baseline and 24 weeks. There were no differences at baseline in demographic or disease-related features between these treatment groups (Table 1), or between each group and the intention-to-treat cohort from which it derived [13]. The mean values for ΔmTSS from baseline to week 24 were 1.0 for patients treated with placebo versus -0.2 for those treated with adalimumab (*P *< 0.001) [13]. Radiographic progression (defined as ΔmTSS > 0.5) from baseline to week 24 was observed in 44 of 152 (29%) patients treated with placebo versus 13 of 144 (9%) treated with adalimumab (*P *< 0.001) [14]. The mean CRP concentration was essentially unchanged during 24 weeks of placebo treatment, whereas with adalimumab the concentration had declined by 63% at week 2 and varied minimally thereafter (Figure 1).

**Table 1:** ADEPT baseline demographics and disease severity characteristics

Characteristic	Placebo (n = 152)	Adalimumab 40 mg every other week (n = 144)
Age (years)^a^	49.4 ± 11.1	47.8 ± 11.9
Male (%)	55.3	56.3
White (%)	94.1	97.2
Body weight (kg)^a^	85.4 ± 16.4	86.1 ± 20.5
Psoriatic arthritis duration (years)^a^	9.3 ± 8.8	9.9 ± 8.3
Psoriasis duration (years)^a^	16.8 ±12.4	17.2 ± 12.1
Modified total Sharp score (0.5 (%)^b^	16.4	11.8
Modified total Sharp score^a^	20.0 ± 36.3	22.3 ± 46.6
Joint erosion score	10.5 ± 20.2	11.4 ± 25.9
Joint space narrowing score	9.5 ± 17.3	10.9 ± 21.8
CRP (0.287 mg/dl (%)^c^	22.4	19.4
CRP (mg/dl)^a^	1.4 ± 1.7	1.4 ± 2.1
Rheumatoid factor-negative (%)	90.1	89.6
Patients taking methotrexate at baseline (%)	51.3	51.4
Tender joint count (0 to 78 joints)^a^	25.8 ± 17.9	23.6 ± 17.4
Swollen joint count (0 to 76 joints)^a^	14.4 ± 11.4	13.6 ± 11.6
HAQ Disability Index (0 to 3)^a^	1.0 ± 0.7	1.0 ± 0.6

HAQ, Health Assessment Questionnaire. *P *> 0.2 for all adalimumab vs. placebo. ^a^Mean ± standard deviation. ^b^Absence of radiographic progression, defined as modified total Sharp score ≤0.5. ^c^Normal C-reactive protein (CRP) by high-sensitivity assay, defined as ≤0.287 mg/dl.

### Univariate analyses of factors potentially associated with radiographic progression

Univariate analyses of the combined placebo-treated and adalimumab-treated groups showed that baseline CRP (whether analyzed as a continuous or a dichotomous variable), baseline mTSS, and baseline JE and JSN scores were each predictive (*P *< 0.05) of radiographic progression (Table 2). Treatment with adalimumab was protective from radiographic progression. The 24-week time-averaged CRP, an indicator of treatment effect, was significantly associated with radiographic progression in this univariate analysis.

**Table 2:** Univariate analyses of predictors for radiographic progression

	Placebo-treated patients			Adalimumab-treated patients			All patients		
			
	OR	95% CI	*P *value	OR	95% CI	*P *value	OR	95% CI	*P *value
Treatment with adalimumab^a^	NA	NA	NA	NA	NA	NA	**0.24**	**0.13, 0.48**	**< 0.001**
CRP (time-averaged)	**1.74**	**1.33, 2.28**	**< 0.001**	1.48	0.99, 2.22	0.058	**1.84**	**1.47, 2.30**	**< 0.001**
CRP (continuous)	**1.40**	**1.12, 1.75**	**0.003**	1.05	0.84, 1.31	0.684	**1.18**	**1.03, 1.35**	**0.017**
CRP > 0.287 mg/dl^a^	**5.50**	**1.59, 19.09**	**0.007**	3.12	0.39, 25.02	0.285	**4.25**	**1.47, 12.24**	**0.007**
CRP (1.0 mg/dl^a^	**3.98**	**1.90, 8.32**	**< 0.001**	**3.51**	**1.08, 11.39**	**0.036**	**3.82**	**2.09, 6.99**	**< 0.001**
CRP (2.0 mg/dl^a^	**4.40**	**2.05, 9.45**	**< 0.001**	1.34	0.34, 5.23	0.676	**3.46**	**1.87, 6.42**	**< 0.001**
Age	1.00	0.97, 1.03	0.954	1.02	0.97, 1.07	0.399	1.01	0.98, 1.04	0.484
RF-positive^a^	1.45	0.46, 4.60	0.530	1.65	0.33, 8.27	0.542	1.41	0.57, 3.49	0.458
Swollen joint count	1.02	0.99, 1.06	0.122	1.01	0.97, 1.06	0.657	1.02	1.00, 1.04	0.100
Tender joint count	1.00	0.98, 1.02	0.741	1.01	0.98, 1.04	0.459	1.01	0.99, 1.02	0.358
mTSS	**1.03**	**1.01, 1.04**	**< 0.001**	1.00	0.995, 1.01	0.365	**1.01**	**1.00, 1.02**	**0.001**
Joint erosion	**1.05**	**1.02, 1.08**	**< 0.001**	1.01	0.99, 1.02	0.490	**1.02**	**1.01, 1.03**	**0.001**
Joint space narrowing	**1.05**	**1.03, 1.08**	**< 0.001**	1.01	0.99, 1.03	0.264	**1.02**	**1.01, 1.04**	**0.001**
Methotrexate (yes)^a^	1.37	0.67, 2.77	0.387	2.15	0.63, 7.33	0.222	1.47	0.82, 2.64	0.201
PsA duration	1.00	0.96, 1.04	0.816	1.03	0.97, 1.10	0.337	1.00	0.97, 1.04	0.896
Psoriasis duration	**0.97**	**0.93, 0.998**	**0.039**	1.03	0.99, 1.08	0.140	0.99	0.96, 1.01	0.277
Weight	1.01	0.99, 1.03	0.450	1.00	0.97, 1.03	0.848	1.00	0.99, 1.02	0.717

CI, confidence interval; CRP, C-reactive protein; mTSS, modified total Sharp score; NA, not applicable; OR, odds ratio; PsA, psoriatic arthritis; RF, rheumatoid factor. All variables are at baseline, except CRP (time-averaged). ^a^Dichotomous variables - those without a footnote annotation are continuous variables.

Univariate analyses were also performed with the placebo and adalimumab groups considered separately, to determine whether they differed in their risk factors for radiographic progression. For placebo-treated patients, the strongest positive associations with radiographic progression (that is, greatest odds ratio (OR)) were observed for baseline CRP as a dichotomous variable (at CRP thresholds > 0.287 mg/dl, ≥1.0 mg/dl and ≥2.0 mg/dl), baseline CRP as a continuous variable, and mean CRP over time (Table 2). Baseline mTSS, JE score, and JSN score were also associated with progression for placebo-treated patients, and baseline psoriasis duration was associated with protection against progression. For adalimumab-treated patients, the only OR that was significantly different from unity was for baseline CRP ≥1.0 (Table 2). Univariate analyses thus demonstrated that inflammation, as indicated by an elevated CRP at baseline or over time, was more strongly associated with radiographic progression for patients who received placebo, than for patients who received adalimumab.

### Relationship between C-reactive protein and radiographic progression

**Figure 2:**
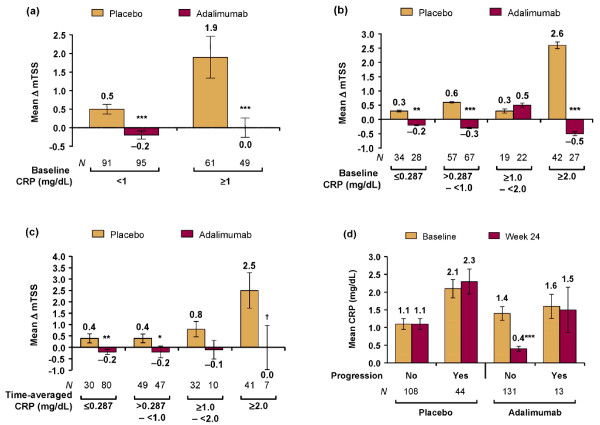
**Mean change in modified total Sharp score for C-reactive protein subgroups**. Changes in modified total Sharp score (ΔmTSS) from baseline to week 24 with standard error were determined for patients grouped *post hoc *by **(a) **baseline C-reactive protein (CRP) categorized as < 1 mg/dl and ≥1 mg/dl, **(b) **baseline C-reactive protein (CRP) categorized as ≤0.287 mg/dl, > 0.287 to < 1 mg/dl, ≥1.0 to < 2.0 mg/dl, and ≥2 mg/dl, and **(c) **24-week time-averaged CRP categorized as ≤0.287 mg/dl, > 0.287 to < 1 mg/dl, ≥1.0 to < 2.0 mg/dl, and ≥2 mg/dl. **(d) **Mean CRP values at baseline and week 24, with standard error, were determined for patients grouped according to whether they had radiographic progression (ΔmTSS > 0.5) between baseline and week 24. Data are observed; except for CRP values in (c), which used the last observation carried forward for missing values. *P < 0.05, **P < 0.01, ***P ≤0.001, †*P *= 0.052 vs. placebo.

Subanalyses were performed to elucidate the relationship between CRP and radiographic progression. At baseline, CRP was < 1.0 mg/dl for 91 of 152 (60%) patients in the placebo arm versus 95 of 144 (66%) in the adalimumab arm (*P *= 0.282). For patients treated with placebo versus adalimumab, the mean ΔmTSS was 0.5 versus -0.2 (*P *< 0.001) for those with baseline CRP < 1.0 and was 1.9 versus 0.0 (*P *< 0.001) for those with baseline CRP ≥1.0 mg/dl (Figure 2a). To assess ΔmTSS across a spectrum of CRP values, patients were analyzed in four categories of baseline CRP. The mean ΔmTSS values were greater with placebo than with adalimumab for three of the four CRP categories, with the greatest difference (2.6 vs. -0.5) observed between the groups with baseline CRP ≥2.0 mg/dl (Figure 2a). For patients with baseline CRP values ≥1.0 to < 2.0 mg/dl, the mean ΔmTSS values were similar for placebo and adalimumab - reflecting the small numbers of patients and the fact that, of the eight greatest ΔmTSS values observed among adalimumab-treated patients, five occurred in this CRP group (with the other three being in patients with baseline CRP ≥2.0 mg/dl). A similar pattern of results was obtained for patients grouped by four categories of mean CRP over time, with the mean ΔmTSS being greatest for the placebo-treated groups with greatest time-averaged CRP, and ≤0 for each adalimumab group (Figure 2c).

These results demonstrate that an elevated CRP was less important as a risk factor for radiographic progression with adalimumab than placebo. Poorly controlled inflammation, however, may have influenced radiographic progression during adalimumab therapy because the eight greatest increases in mTSS values observed during adalimumab therapy occurred in patients with baseline CRP ≥1.0 mg/dl. Moreover, the mean CRP concentration for adalimumab-treated progressors (n = 13) was essentially unchanged by treatment (Figure 2c), whereas for adalimumab-treated nonprogressors (n = 131) the mean concentration was markedly reduced by treatment (Figure 2d).

### Cumulative probability plots of ΔmTSS by C-reactive protein concentration at baseline

**Figure 3:**
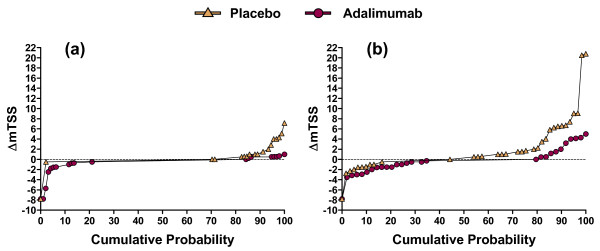
**Cumulative probability plots of mean change in modified total Sharp score**. Cumulative probability plots displaying the changes in modified total Sharp score (ΔmTSS) from baseline to week 24 were generated for **(a) **patients with baseline C-reactive protein (CRP) < 1.0 mg/dl and **(b) **patients with baseline CRP ≥1.0 mg/dl. For each CRP category, the placebo curve is above the adalimumab curve, indicating greater radiographic progression.

To further clarify the relationship between baseline CRP and joint damage, radiographic outcomes were evaluated with cumulative probability plots of ΔmTSS from baseline to week 24. The placebo curve was above the adalimumab curve (indicating a worse overall radiographic outcome with placebo) for patients with baseline CRP < 1.0 mg/dl (Figure 3a) and, to a greater degree, for patients with baseline CRP ≥1.0 mg/dl (Figure 3b). With adalimumab, radiographic progression occurred almost only among patients with baseline CRP ≥1.0 mg/dl, and was never as great as in the most severe cases observed with placebo. Improvement in mTSS (seen in the left-hand side of a curve) was more prominent among adalimumab-treated patients, especially among those with baseline CRP ≥1.0 mg/dl. These cumulative probability plots demonstrate that the radiographic efficacy of adalimumab, as measured in ADEPT, represented reduced worsening and greater improvement of mTSS.

### Multivariate analysis of baseline factors associated with radiographic progression

A multivariate logistic regression analysis was performed to determine whether CRP or other baseline factors identified in the univariate analyses were independently associated with radiographic progression. The analysis considered all patients as a single group, and included the baseline variables that were statistically significant in the univariate analyses, as well as selected demographic variables (Table 3). The results demonstrated that an elevated baseline CRP concentration was an independent predictor for radiographic progression, whether CRP was assessed as a continuous variable (OR = 1.16, 95% confidence interval (CI) = 1.01 to 1.33, *P *= 0.040) or a dichotomous variable (for CRP > 0.287: OR = 4.31, 95% CI = 1.42 to 13.09, *P *= 0.010; for CRP ≥1.0: OR = 3.28 95% CI = 1.66 to 6.51, *P *< 0.001; and for CRP ≥2.0: OR = 2.29 95% CI = 1.12 to 4.69, *P *= 0.023). An elevated mTSS at baseline was the only other independent predictor of radiographic progression, but the increase in risk was small (OR = 1.01, 95% CI = 1.00 to 1.02, *P *= 0.002). Treatment with adalimumab was a strong independent predictor of decreased radiographic progression, with a nearly fivefold reduction in frequency of radiographic progression (OR = 0.21, 95% CI = 0.10 to 0.42, *P *< 0.001).

**Table 3:** Multivariate analysis of predictors for radiographic progression

Variable	OR	95% CI	*P *value
Treatment with adalimumab^a^	0.21	0.10, 0.42	< 0.001
CRP (continuous)	1.16	1.01, 1.33	0.040
CRP > 0.287 mg/dl^a^	4.31	1.42, 13.09	0.010
CRP (1.0 mg/dl^a^	3.28	1.66, 6.51	< 0.001
CRP (2.0 mg/dl^a^	2.29	1.12, 4.69	0.023
Modified total Sharp score	1.01	1.00, 1.02	0.002

CI, confidence interval; CRP, C-reactive protein; OR, odds ratio. Separate multivariate analyses were performed for CRP (continuous), CRP > 0.287 mg/dl, CRP ≥1.0 mg/dl and CRP ≥2.0 mg/dl. For non-CRP variables, results are from the analysis with CRP > 0.287 mg/dl. All multivariate analyses also included baseline age, rheumatoid factor (yes/no), tender joint count, swollen joint count, and methotrexate use (yes/no) as variables; *P *> 0.05 for each. ^a^Dichotomous variables - those without a footnote annotation are continuous variables.

## Discussion

The goals of treatment in patients with PsA are to control symptoms and signs of inflammation and to prevent progression of joint damage. The present *post hoc *analysis has extended previous studies of joint damage in PsA [6,13,14] by identifying factors that independently predicted radiographic progression in a randomized, placebo-controlled study of PsA (ADEPT). Furthermore, it determined whether these factors had similar effects for patients treated with adalimumab versus those treated with placebo. The principal finding, obtained from a multivariate linear regression analysis, was that an elevated baseline CRP concentration was the dominant independent risk factor for radiographic progression in PsA. Treatment with adalimumab was the dominant predictor for reduced radiographic progression.

Univariate analyses demonstrated that elevated baseline CRP was strongly associated with radiographic progression only for placebo-treated patients. In contrast, while almost no radiographic progression occurred among adalimumab-treated patients with baseline CRP < 1.0 mg/dl, those with baseline CRP ≥2.0 mg/dl had the best overall radiographic outcomes; that is, the lowest mean ΔmTSS. These results indicate that the baseline CRP concentration was a predictor of which patients with PsA were at risk for subsequent joint damage, that adalimumab was effective for preventing most joint damage in PsA, and that the greatest radiographic benefit from adalimumab occurred for patients with the greatest baseline CRP concentrations.

Cumulative probability plots demonstrated that adalimumab prevented nearly all radiographic progression among patients with baseline CRP < 1.0 mg/dl and, compared with placebo, most radiographic progression among patients with baseline ≥CRP 1.0 mg/dl (Figure 3). They also illustrated that the mTSS did not increase for a substantial portion of placebo-treated patients, including some with baseline CRP ≥1.0 mg/dl. This finding was not attributable to concomitant use of methotrexate [14] and was too frequent to have been caused only by spontaneous improvements in clinical arthritis activity [13]. Instead, the absence of radiographic progression in some patients probably reflects true disease heterogeneity, plus several quantitative factors - including the tendency for PsA to cause radiographically detectable joint damage less frequently than RA, the limited duration of placebo-controlled observation in ADEPT (24 weeks), and the ability of radiographs to detect only a portion of total joint damage [21].

The magnitude of radiographic benefit observed here with adalimumab is probably a low estimate compared with what might have been observed if adalimumab and placebo had been compared over a longer period of time. Consistent with this expectation, adalimumab inhibited radiographic progression in patients from ADEPT to 48 weeks [14] and to 2 years [22]. Although baseline CRP was < 1.0 mg/dl in a smaller portion of patients treated with placebo (60%) than adalimumab (66%; *P *= 0.282) in ADEPT, the overall radiographic efficacy of adalimumab [13] cannot be explained by this difference because mean ΔmTSS was significantly lower with adalimumab, regardless of whether baseline CRP was < 1.0 mg/dl or ≥1.0 mg/dl.

This analysis also found that radiographic joint damage at baseline was an independent predictor of radiographic progression. The magnitude of this association was small in the multivariate analysis, both in absolute terms and when compared with the results for baseline CRP variables (Table 3). Baseline joint damage also predicted progression of damage in a previous observational cohort study [6]. In that study, tender and swollen joints were identified as independent predictors of radiographic progression [6]; in the current study, however, TJC and SJC were predictive of progression only in the univariate analysis, not in the multivariate analysis. This disparity between studies may be related to the finding that CRP was a stronger predictor of joint damage than were the physical examination-based indicators of arthritic inflammation. The disparity may also be related to the greater joint counts at baseline in ADEPT (mean values of 24 tender joints and 14 swollen joints), compared with the observational cohort study [6].

The present study focused mainly on evaluating baseline measures as predictors of joint damage. It also examined the role of treatment effect, by assessing patients in terms of their 24-week time-averaged CRP. For placebo-treated patients, the association between ΔmTSS and time-averaged CRP was similar to that observed between ΔmTSS and baseline CRP, probably because an elevated CRP tends to remain elevated without treatment (Figure 2d). For adalimumab-treated patients, the mean ΔmTSS was inhibited regardless of the concentration of time-averaged CRP. Adalimumab thus appeared to inhibit radiographic progression not only by reducing inflammation, but also via effects that were not entirely dependent on control of inflammation. These findings are consistent with evidence that TNF antagonists can directly inhibit the osteoclast pathway in patients with PsA [23,24], and with radiographic outcomes observed in patients with RA [21,25,26]. Further research is needed to understand variations in responses to TNF antagonists, not only for individual patients, but also with respect to destructive versus proliferative joint disease in PsA.

The main limitation of the analyses presented here is that they were designed *post hoc*. ADEPT is a unique resource because it is the only randomized controlled trial of a TNF antagonist in PsA that continued placebo therapy for 24 weeks without an escape option. The 24-week duration limited the ΔmTSS in placebo-treated patients to a mean value of 1.0, however, and did not allow radiographic efficacy to be assessed in the full study population after an initial period of therapy. The number of adalimumab-treated patients with radiographic progression was small, which precluded subset analyses in this group. The mTSS used for ADEPT was modified for PsA by including the distal interphalangeal joints, but it only measured erosions and joint space narrowing and only in peripheral joints. Other PsA-related radiographic findings were assessed in ADEPT, such as juxtaarticular periostitis and pencil-in-cup. They changed by amounts that were too small to warrant their inclusion in the present analysis [14].

## Conclusions

We have shown that an elevated CRP concentration at baseline was an independent predictor of radiographic progression for patients with PsA. This risk relationship was strong only for patients treated with placebo. Treatment with adalimumab rapidly reduced the mean CRP concentration, and was an independent predictor of reduced radiographic progression in PsA. Adalimumab inhibited radiographic progression across the spectrum of baseline or time-averaged CRP, doing so by preventing nearly all progression in patients with baseline CRP < 1.0 mg/dl and greatly limiting it in others. These analyses demonstrate that patients with the greatest elevations in baseline CRP were at greatest risk for joint damage if untreated, and were the ones who derived greatest radiographic benefit when treated with adalimumab.

## Abbreviations

ADEPT: Adalimumab Effectiveness in PsA Trial; BSA: body surface area; CI: confidence interval; CRP: C-reactive protein; ΔmTSS: change in modified total Sharp score; JE: joint erosion; JSN: joint space narrowing; mTSS: modified total Sharp score; OR: odds ratio; PsA: psoriatic arthritis; RA: rheumatoid arthritis; SJC: swollen joint count; TJC: tender joint count; TNF: tumor necrosis factor.

## Competing interests

DDG has received consulting fees and honoraria from Abbott Laboratories, Amgen, Bristol-Myers Squibb, Centocor, Roche, UCB, Schering, and Wyeth, and has received unrestricted research and education funds from Abbott, Amgen, Schering, and Wyeth. PJM has received research grants and consultant and/or speakers' bureau honoraria from Abbott Laboratories, Amgen, BiogenIdec, Bristol Myers Squibb, Centocor, Genentech, Pfizer, Roche, UCB, and Wyeth. EHSC has served as consultant, participated in speakers' bureaus, and received research grants from Abbott Laboratories, Schering-Plough, Wyeth, Pfizer, GlaxoSmithKline, UCB, Eli Lilly, Jazz Pharmaceuticals, Boehringer Ingelheim, Pierre Fabre Medicament, Chelsea Therapeutics, and Roche. CTR has received consulting fees and honoraria from Abbott Laboratories, Centocor, Wyeth, and Biogen. RJP and EHS are employees of Abbott Laboratories, and both own Abbott stock and hold Abbott stock options.

## Authors' contributions

DDG, PJM, EHSC, and CTR (with other academic experts and members of Abbott Laboratories) designed the ADEPT clinical study. EHS and DDG designed analyses for the present report, and RJP conducted the analyses. DDG, PJM, EHSC, and CTR were involved in acquisition of data. All authors participated in analysis and interpretation of the data. EHS and DDG drafted the manuscript. All authors reviewed and approved the final content of the submitted manuscript.
